# *Agaricus bisporus* Wild Mushroom Extract as Lectin Source for Engineering a Lactose Photoelectrochemical Biosensor

**DOI:** 10.3390/bios13020224

**Published:** 2023-02-03

**Authors:** André O. Santos, Vanessa E. Abrantes-Coutinho, Simone Morais, Thiago M. B. F. Oliveira

**Affiliations:** 1 Centro de Ciência e Tecnologia, Universidade Federal do Cariri, Juazeiro do Norte 63048-080, CE, Brazil; 2 REQUIMTE-LAQV, Instituto Superior de Engenharia do Porto, Instituto Politécnico do Porto, Rua Dr. António Bernardino de Almeida, 431, 4249-015 Porto, Portugal

**Keywords:** *Agaricus bisporus* mushroom, lectins, lactose analysis, photoelectrochemical biosensor

## Abstract

*Agaricus bisporus* mushroom biomass contains a lectin, ABL, with remarkable specificity for lactose biorecognition; in this work, this feature was explored to develop a photoelectrochemical biosensor. The high lectin activity found in saline extracts of this macrofungus (640 HU mL^−1^), even at critical pH values (4–10) and temperatures (20–100 °C), allowed its direct use as an ABL source. Theoretical and experimental evidence revealed favorable electrostatic and biocompatible conditions to immobilize ABL on a poly(methylene blue)/fluorine-doped tin oxide-coated glass platform, giving rise to the ABL/PMB/FTO biosensor. The conducting polymer added further photoactivity to the device, allowing the identification of lectin–carbohydrate interactions with even greater sensitivity. The dose–response curves studied by electrochemical impedance spectroscopy showed a sigmoidal profile that was well-fitted by Hill’s equation, expanding the working dynamic range (15–540 nmol L^−1^ lactose; 20.2 pmol L^−1^ detection limit) and avoiding undesirable sample dilution or preconcentration procedures. Under the optimized photoelectrochemical conditions, the ABL/PMB/FTO biosensor showed remarkable signal stability, accuracy, specificity, and selectivity to analyze lactose in commercial food products. This research raises interest in ABL-based biosensors and the added value of the crude *Agaricus bisporus* extract toward the development of greener and more sustainable biotechnological approaches.

## 1. Introduction

Macro- and micro-fungi are considered important sources of lectins, i.e., proteins of nonimmune origin with carbohydrate-specific domains [1,2]. The interaction between lectins and carbohydrates or glycoconjugates occurs reversibly and maintains the protein’s native conformation; this feature is among the main technological attributes of these biomolecules [2]. Despite the rich fungal diversity distributed all over the world, wild mushrooms stand out among the main sources of lectins, contributing to approximately 80% of the identified structures [1,3]. The first study on mushroom lectins dates back to 1910, when the toxicological properties of *Amanita muscaria* were investigated [4]. Over time, these species have captured more attention for their immeasurable nutritional and pharmacological value, showing antiproliferative, immunomodulatory, and antibacterial properties associated with lectin activity [1,2,4,5]. The presence of these macromolecules (generally 15–90 kDa molecular weight [6]) in the fruiting bodies and vegetative mycelia of several mushrooms has been reported, including those expressed by the genera *Agaricus*, *Coprinus*, *Lentinus*, *Ganoderma*, *Pleurotus*, *Pycnoporus*, *Schizophyllum*, *Trametes*, and *Volvariella* as representative examples [1,4,5]. Mushroom lectins are different from each other and from those found in plants and animals in terms of their amino acid sequence, number of subunits, affinity for different carbohydrates/glycoconjugates, and chain stability at different pH levels and temperatures [7]. Hence, each lectin has unique physicochemical and biochemical properties that can lead to different results, new insights, and discoveries.

Research conducted with cells, extracts, and metabolites isolated from *Agaricus bisporus* (also called white mushroom, button mushroom, or champignon) has attested to its great biotechnological value [8,9,10,11]. Its genomic sequence was unveiled in 2012, and since then, only one lectin has been registered in GenBank (ABL; ID XP_006455249) [12]. In the mid-1990s, two ABL cDNA sequences were cloned and the tetrameric arrangement of their native structures was deciphered [13]. The four constituent subunits are isoforms that behave like glycoproteins, with similar carbohydrate specificities and molecular weights (~16 kDa). Regarding carbohydrate-binding modules (CBMs), the ABL genome includes: (i) CBM1 and CBM5, which justify its affinity for polymeric carbohydrates, such as cellulose and lignin; (ii) CBM13 and CBM20, which interact with non-branched oligosaccharides (*n* ≥ 4 sugar units); and CBM 21 and CBM35, which are responsible for the interaction with less complex carbohydrate molecules [12]. Lectins with the amino acid sequence CBM13 also have affinity for disaccharides and trisaccharides derived from pyranose (association constant ~ 1 × 10^2^–1 × 10^3^ M^−1^ s^−1^) [12,14], making them highly versatile for screening sugars by regioselective interactions.

ABL is classified as a hololectin, has high affinity for galactose-*β*-1,3-*N*-acetylgalactosamine (Gal-*β*-1,3-GalNAc; also known as T-antigen disaccharide) and galactose-*β*-1,3-*N*-acetylglucosamine, but does not bind to monosaccharides [4,15]. There is theoretical and experimental evidence proving that ABL also recognizes lactose (*β*-D-galactopyranosyl-(1→4)-D-glucose) with intriguing skill [15,16]. This feature is very attractive in bioassays that require high specificity and selectivity to quantify this disaccharide in complex samples, although related studies are seldom [2]. It is worth remembering that a diet rich in lactose-based products guarantees important vitamins and minerals for our health, but people with lactose intolerance face serious gastrointestinal and extra-intestinal problems [17]. Accurate quality control of natural products and commercial formulations is very important for adherence to lactose-free diets, as well as for devising more appropriate nutritional and therapeutic strategies [18].

Traditional procedures based on polarimetry, gravimetry, potentiometry, infrared spectroscopy, liquid/gas chromatography, and enzymatic trials are among the alternatives recognized by analytical standard agencies for lactose monitoring [19,20,21]. In general, these options require analyte extraction or derivatization and, sometimes, deal with sensitivity and specificity problems, thwarting their widespread adoption and demanding more research and innovative ideas in this field [20,22,23]. Biosensors developed from fungal extracts associated with suitable (photo)electrochemical transducers make up a narrow technological niche under study to overcome these challenges [24]. This initiative avoids laborious and time-consuming steps for protein purification, preserves the conformation and reactivity of native structures, and dramatically reduces device production costs [24,25]. Photoelectrochemical methods employ light as an excitation source, generating an amplified analytical signal compared with electrochemical or chemiluminescent ones [26]. Photoactive semiconductors also catalyze the redox reactions of various (in)organic substances, reducing the electrochemical potential and energy consumption required to operate the (bio)sensors. These devices are also simple to build, inexpensive, and can be miniaturized for on-site and point-of-care analyses [26,27,28].

The synthesis and improvement of photoactive semiconductors are in full development [29] and, although less explored, polyphenothiazines derivatives are attractive compounds for electronic devices, as their heteroatoms (i.e., nitrogen and sulfur) and π-bond arrangements confer a charge-transfer state favorable to the mobility of electrons and holes. Starting from this assumption, a novel, highly sensitive, and specific lactose photoelectrochemical biosensor is proposed here using a poly(methylene blue) (PMB)/fluorine-doped tin oxide-coated glass (FTO) platform, after its prior modification with an ABL-enriched protein extract. This extract was successfully recovered from wild mushrooms (*Agaricus bisporus*) from the Araripe National Forest, Ceará State, Brazil. The ABL activity (purified and dispersed in mushroom extract) was extensively characterized by two-dimensional gel electrophoresis, UV-visible spectroscopy, hemagglutination assays, and voltammetric techniques. Complementary data on the morpho-functional properties of the (bio)materials were obtained by infrared spectroscopy, thermal analysis, electron microscopy, and impedance. The construction, detection, and analytical performance of the biosensor were also thoroughly studied and discussed to ascertain its feasibility for lactose biorecognition. The proposed photoelectrochemical biosensor was able to quantify lactose in commercial food products with great accuracy, sensitivity, and specificity.

## 2. Materials and Methods

### 2.1. Chemicals

Anhydrous lactose was purchased from Neon, Brazil. Protein extraction from *Agaricus bisporus* biomass was performed with neutral solutions of tris(hydroxymethyl)aminomethane, monobasic/dibasic sodium phosphate, and sodium chloride, all supplied by LabSynth, Brazil. Other reagents used to treat/preserve protein extracts (2-mercaptoethanol and polyvinylpyrrolidone), in the composition of chromatographic eluents (acetic acid/sodium acetate and glycine), electrolytic solutions (methylene blue and sodium borate), and electrophoretic tests (acrylamide/bisacrylamide, sodium dodecyl sulfate, ammonium persulfate, glycerol, methanol, 1,2-bis(dimethylamino)ethane, dyes, and markers) were purchased from Sigma-Aldrich, Brazil. Chemicals were of analytical grade and used without further purification. All solutions were prepared with ultra-purified water (*ρ* = 18 MΩ cm^−1^) from a Millipore Milli-Q system—Brazil.

### 2.2. Protein Extraction and Purification

The mushrooms used as feedstock were sampled in the Araripe National Forest, Ceará State, Brazil. The fungal material was collected in plastic bags and properly sterilized and identified, followed by biomass refrigeration at 5 °C until the extraction of the protein content. The feedstock was dehydrated in a microprocessor forced-air oven (Sterilifer, São Caetano do Sul, Brazil) kept at 40 °C for 72 h. After converting the resulting biomass into powder, solid–liquid extraction processes were performed in Tris-HCl buffer, phosphate buffer, or NaCl solution, all prepared at 0.15 mol L^−1^ and adjusted to pH 7.0. Solid–liquid partition processes (1:10%, *w*/*v*) were conducted for 12 h using an orbital shaker (Tecnal, Ourinhos, Brazil) set at 1000 rpm and keeping the samples at 20 °C. Then, the suspension was filtered slowly through quantitative paper (2.5 microns; Whatman, Maidstone, UK) to remove insoluble residues, and the filtrate was used as the ABL-enriched protein extract.

To verify the relevance of the protein extract for analytical purposes, a purified ABL fraction was also isolated for data comparison. In that regard, the mushroom extract was saturated with ammonium sulfate (80%, *w*/*v*) to recover the protein content by precipitation. The biomaterial was separated by centrifugation at 10,000 rpm (Sieger, Pernambuco, Brazil) for 10 min, redissolved in 10 mL of distilled water, and later dialyzed against the same solvent for 48 h. The resulting mixture was applied to a DEAE–cellulose anion exchange column chromatograph (2.5 cm × 20 cm; Sigma Aldrich, St. Louis, MO, USA), previously equilibrated and eluted with 10 mmol L^−1^ of Tris-HCl buffer (pH = 8.2). The adsorbed fraction was post-eluted in the same solvent, but containing 0.1–0.5 mol L^−1^ NaCl. This eluate was subjected to a second purification step by affinity chromatography, performed with a Sepharose 4B column (5.0 cm × 20 cm; Sigma Aldrich) previously equilibrated with 10 mmol L^−1^ Tris-HCl buffer (pH = 7.0). The retained fraction was recovered with 10 mmol L^−1^ glycine buffer (pH = 9.0), dialyzed, and used as the purified portion of protein.

### 2.3. Protein Activity Tests

The total protein content of the mushroom extracts was evaluated by the Bradford spectrophotometric method [30], which is based on the interaction between amino acids of the peptide chain and Coomassie brilliant blue dye (G-250). For each 100 µL of sample, 2.5 mL of Bradford’s reagent was added, consisting of 0.05 g of G-250, 25 mL of 95% ethanol, and 50 mL of 85% orthophosphoric acid, homogenized in 500 mL of aqueous solution. The compound formed was determined at its maximum absorption at 595 nm.

Lectin activity was confirmed by hemagglutination assays performed with rabbit erythrocytes [31]. The procedure consisted of serial 2-fold dilutions of ABL-based extract (640 HU mL^−1^) in 2.0% (*v*/*v*) blood cell suspension prepared with PBS (pH = 7.2), yielding 100 µL of mixture. The results were evaluated after 1.0 h of incubation at 20 °C, allowing complete sedimentation of the negative control. An analogous procedure was used to investigate the resistance of protein activity at different pH values (4–10) and temperatures (20–100 °C), as well as to assess the lectin specificity by hemagglutination inhibition tests performed in the presence of different carbohydrates: glucose, fructose, maltose, lactose, sucrose, cellulose, and starch. The hemagglutinating activity titer was estimated by the minimum amount of lectin (hemagglutination units; HU) that showed cell agglutination, while its specific activity was expressed in hemagglutinating units per protein mass (HU mg^−1^). All procedures conducted with rabbits were authorized by the Institutional Animal Ethics Committee (Protocol No. 14/2022—CEUA/UFCA) and followed international guidelines—EU Directive 2010/63; Council Directive 86/609/EEC; OJL 358, December 18, 1986; and NIH Guide for Care and Use of Laboratory Animals.

### 2.4. Characterization of (Bio)materials and Analytical Devices

ABL extraction and purification were conducted by sodium dodecyl sulfate–polyacrylamide gel electrophoresis (SDS-PAGE). For this, the extracts and isolated proteins were diluted in a solubilizing solution containing 1.0% SDS, 0.02% bromophenol, and 1.0% *β*-mercaptoethanol dispersed in running buffer (0.25 mol L^−1^ Tris-HCL and 1.92 mol L^−1^ glycine; pH = 6.8), and the resulting mixtures were heated at 80 °C for 10 min before electrophoresis. The stacking gel contained 3.5% acrylamide in 0.5 mol L^−1^ Tris-HCl buffer (pH = 6.8), while the separating gel consisted of 12% acrylamide and 0.01% SDS, dispersed in 1.5 mol L^−1^ Tris-HCl buffer (pH = 8.8). SDS-PAGE experiments were performed in a 170 mm × 150 mm × 1.5 mm slab gel system, which was calibrated with bovine serum albumin (BSA; 66 kDa) and Concanavalin A (ConA; 28 kDa) as molecular mass markers. The separation process was controlled by a 300STD electrophoresis power supply (GSR, São Paulo, Brazil) using 200 V tension, 25 mA current, and 20 W power. Gels were stained with 0.1% G-250.

The (photo)electrochemical studies on the protein–carbohydrate interactions were performed with a PGSTAT 128N potentiostat/galvanostat (Metrohm—Autolab, Utrecht, The Netherlands) equipped with an FRA32M impedance module and controlled by NOVA 2.0 software. This workstation was operated with a conventional three-electrode electrochemical cell: (i) ABL extract-based biosensor as a working electrode, (ii) a platinum auxiliary electrode, and (iii) a Ag/AgCl/Cl^−^ (3.0 mol L^−1^ KCl) reference electrode; all connected by copper alligator clips. The photoexcitation of the biosensor’s surface was promoted by a blue light-emitting diode (470 nm LED with 10 W power). Different control parameters, such as the peak current (*I_p_*), peak potential (*E_p_*), half-peak width (Δ*E_p/2_*), and properties associated with charge-transfer resistance (*R_ct_*) were monitored by cyclic voltammetry (CV) and electrochemical impedance spectroscopy (EIS).

The morphological characteristics of the (bio)materials were evaluated by scanning electron microscopy (SEM; Tescan Vega 3, Japan). The thermal stability and reaction enthalpy data were obtained by differential scanning calorimetry (DSC; TG/DTA—60H, Shimadzu, Kyoto, Japan). Fourier-transform infrared spectroscopy (FTIR; Cary 630, Agilent, Santa Clara, CA, USA) information was used to observe the vibrational modes of functional groups present in the protein structure. UV-visible spectroscopy (Cary 50, Varian, Palo Alto, CA, USA) was also used for qualitative and quantitative tests of protein activity.

### 2.5. Semi-Empirical Studies and Molecular Modeling

Theoretical experiments on lectin arrangement and reactivity were designed from the structure available in the Protein Data Bank—PDB 1Y2X. The cartesian coordinates, atomic radii, and partial charges were obtained by the pdb2pqr APBS package [32]. The partial charge distribution according to pH was evaluated by DelphiPKa [33], assuming the presence of 0.15 mol L^−1^ NaCl in the medium. The electrostatic potentials of the macromolecule in alkaline medium were calculated by Delphi [34], maintaining the same saline condition mentioned earlier. VMD software [35] was used to generate 3D lectin images and perform additional analyses.

### 2.6. Biosensor Construction

For the biosensor design, an FTO electrode was initially modified with PMB [36] by electrodeposition in a 0.1 mmol L^−1^ methylene blue solution prepared in 100 mmol L^−1^ sodium borate buffer (pH = 9.0). This process was assisted by CV, polarizing the electrode between −0.3 and 0.9 V for 35 cycles at 50 mV s^−1^. The stabilization/activation of PMB/FTO were performed in the same buffer solution, but with the electrode polarization interrupted after 15 potential cycles. Then, 10 µL of the ABL extract or purified protein solution (1.0 mg mL^−1^) were deposited on PMB/FTO by drop-coating, followed by solvent evaporation for 24 h at room temperature. The resulting biosensor was defined as ABL/PMB/FTO.

### 2.7. (Photo)electrochemical Assays and Data Handling

EIS was used to quantify lactose with ABL/PMB/FTO, so the impedimetric measurements were analyzed by Nyquist diagrams recorded between 100 mHz and 100 kHz frequency, setting the modulation amplitude at 5 mV and using 0.1 mmol L^−1^ K_3_[Fe(CN)_6_] as the redox probe. Analytical curves were constructed from the relationship between the phase angle and carbohydrate concentration, employing the following mathematical expression adapted from the “Hill equation” to fit the dose–response data [37]:

(1) $$\Delta \theta =\Delta \theta_{max}\cdot \frac{{\left[lactose\right]}^{n}}{K_{D}^{n}{+[lactose]}^{n}}$$

In the above equation, Δθ is the percentage difference between the sample and blank phase angles, $\Delta \theta_{max}$ is the maximum percentage difference in the same variables described before, $K_{D}$ represents the apparent dissociation constant of the ABL–lactose complex, and n is Hill’s coefficient. The limits of detection (LOD) and quantification (LOQ) were calculated from the resulting regression equation coefficients recorded in the smallest linear range studied [38]. Possible charge-transfer resistance oscillations recorded for lactose analysis (1.0 × 10^−8^ mol L^−1^) in the presence of different carbohydrates (glucose, fructose, maltose, sucrose, cellulose, and starch) were used as indicative of analytical interference. The method’s precision was analyzed by intraday (n = 10) and interday (n = 5) repeatability tests using 1.0 × 10^−8^ mol L^−1^ lactose, and the results are reported as the relative standard deviation (RSD; %). The reproducibility tests followed a similar protocol, but used different devices (n = 3). All assays were performed in triplicate.

### 2.8. Real Samples Analysis

The photoelectroanalytical method developed was tested for lactose quality control in commercial products of skimmed milk, lactose-free milk, and sweetener. The high specificity achieved with ABL/PMB/FTO toward the analyte allowed the direct application of the procedure, avoiding conventional laborious sample treatment steps to eliminate excipients. The results were validated by comparing the values with those obtained by the standard spectrophotometric method for quantifying reducing sugars based on the reaction with 3,5-dinitrosalisylic acid [39].

## 3. Results and Discussion

### 3.1. Lectin Extraction and Characterization

ABL’s solubility in aqueous media is favored when electrostatic repulsions overcome hydrophobic interactions between protein chains; thus, proper solvent selection is paramount. The lectin peptide chain contains a sequence of 142 amino acids [12], predominantly threonine (11.27%), glycine (9.15%), valine (8.45%), asparagine (7.75%), and arginine (7.04%), which establish hydrogen bonds and/or Van der Waals interactions with polar solvents, allowing the extraction of ABL from fungal biomass without any additives. A total protein content of 0.21 mg mL^−1^ was retrieved from 10 g of *Agaricus bisporus* using pure water at 20 °C. Even so, more appreciable protein concentrations (1.42–2.34 mg mL^−1^) were obtained with NaCl solution, phosphate, and Tris–HCL buffer, all prepared at 0.15 mol L^−1^ and adjusted to pH = 7. Changes in ionic strength control the salting-in and salting-out processes of proteins in aqueous media because they influence the solvation and hydrophobicity conditions of the macromolecules [40]. Normally, native proteins are associated with each other in fungal biomass, compromising their solubility in water and other ordinary solvents. Hofmeister’s theory explains that chaotropic ions can revert that condition by destabilizing hydrogen bond networks between water molecules, along with hydrophobic interactions between secondary protein structures, enhancing biomolecules’ solubilities [40,41,42]. In addition, larger ions have a less structured hydration shell and are more hydrophobic [41], so they can establish stronger interactions with strategic sites scattered along the peptide chain, aiding in protein solubilization and extraction. In this work, the extraction process performed with 0.15 mol L^−1^ Tris–HCL buffer (pH = 7) was more satisfactory, obtaining a 2.34 mg L^−1^ protein yield.

**Figure 1:**
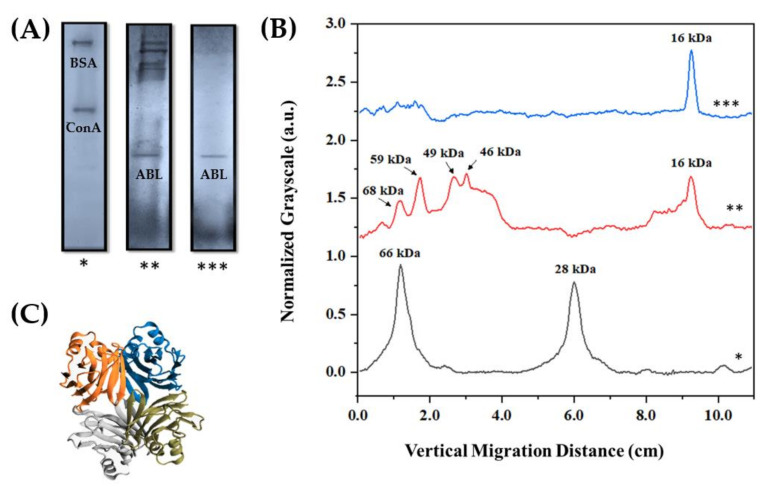
(**A**) Molecular weight estimation of protein residues performed by SDS–PAGE using bovine serum albumin (BSA; 66 kDa) and Concanavalin A (ConA; 28 kDa) as calibration markers, along with a (**B**) theoretical scale based on the vertical migration distance of the electrophoretic bands. The exposed lanes correspond to experimental data obtained with molecular markers (*), crude extract (**), and purified lectin (***) from *Agaricus bisporus* biomass. (**C**) Three-dimensional structure of lectin, highlighting its four isoformic subunits.

Mushrooms secrete several proteins that can be identified in a variable number of subunits according to the chosen extraction conditions. In the extract of *Agaricus bisporus*, several substances of great biotechnological potential can be found [10,12], justifying the variety of electrophoretic bands observed in this study (Figure 1A). Most records referred to macromolecules with molecular weights ranging from 46 kDa to 68 kDa (Figure 1B), but there was another prominent band with a stationary position at 16 kDa, associated with the four ABL isoforms (Figure 1C) as they had relatively close isoelectric points; 5.53 ≤ pI ≤ 6.70 [15,43]. Lectins with molecular weights similar to that of ABL have also been identified in *Agaricus arvensis*, *Agaricus blaezei*, *Agaricus campestris*, and *Agaricus edulis* [44]. The same electrophoretic pattern of ABL was observed for the crude extract and purified fraction of this protein, indicating that the chromatographic procedure adopted was efficient for isolating it. In contrast, the long time required to isolate and purify this lectin, in addition to the reduction in hemagglutinating activity (80 HU mL^−1^) compared with that observed in the crude extract (640 HU mL^−1^), showed that the use of the latter may be more advantageous for developing analytical devices with greater sensitivity.

**Figure 2:**
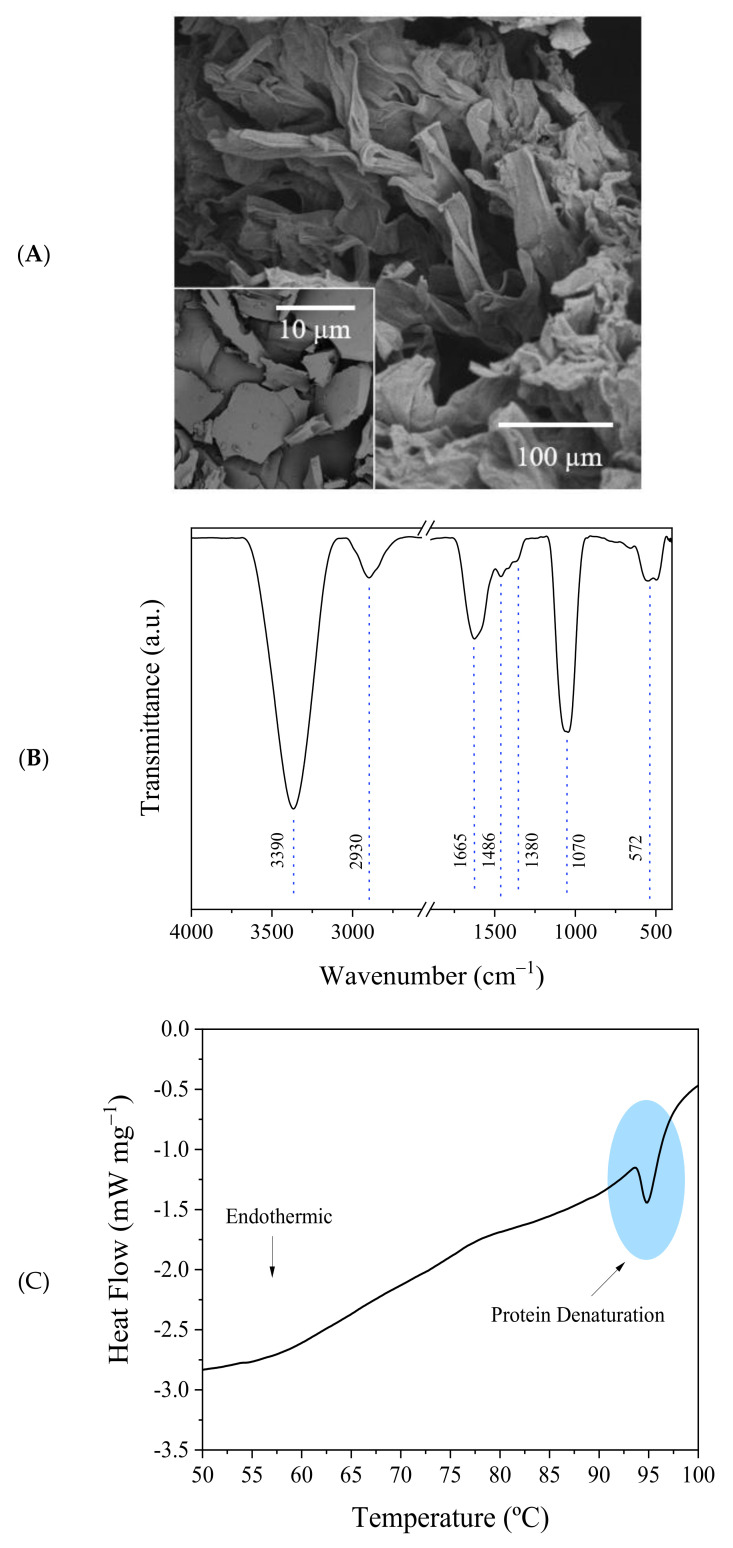
(**A**) Scanning electron micrographs of *Agaricus bisporus* biomass and isolated lectin microcrystals (inset) recorded at 500- and 3000-times magnification, respectively. (**B**) FTIR spectrum and (**C**) differential scanning calorimetry data obtained for the lectin.

It is very likely that the ease of extracting ABL also has a strong relationship with the morphological organization of its natural source. The scanning electron micrograph of dehydrated *Agaricus bisporus* biomass clearly exposes its randomly arranged hyphae, consisting of matted microfilaments (Figure 2A), which is a striking textural feature in many higher fungi [45]. These structures have a high surface area, facilitating the extraction of proteins by solid–liquid partitioning processes. After metalizing the solid referring to the purified ABL, crystalline microflakes with heterogeneous geometry were obtained with size ≈ 45 ± 0.80 µm^2^ and width <0.50 ± 0.04 µm (inset in Figure 2A). ABL purification also made its predominantly *β*-sheet protein (≈79% per monomer) FTIR pattern even more evident, with a moderate absorption band at 1620–1670 cm^−1^ (Figure 2B). The strong and broad peak at 3390 cm^−1^ was attributed to O‒H and N‒H stretching vibrations, while the band around 2930 cm^−1^ was ascribed to aliphatic C‒H stretching. The set of peaks with different intensities between 1250 and 1750 cm^−1^ is characteristic of the amide I-III vibrational modes of the lectin backbone, with emphasis on C=O asymmetric stretching (1665 cm^−1^), N=N and N=O double bond stretching (1486 cm^−1^), and C‒H deviational vibration (1380 cm^−1^). The well-defined peak at 1070 cm^−1^ was attributed to C‒O‒H deformation, and the weak band at 572 cm was related to S‒S stretching vibration arising from disulfide bonds [46]. The good resolution of the FTIR spectrum also confirms the success of lectin purification, allowing reliable comparison tests with the protein extract to be performed.

### 3.2. Lectin Activity Threshold

Studying the limiting conditions of lectin activity in the ABL extract is essential to find ideal conditions for the possible biotechnological application of this material. Therefore, hemagglutinating capacity tests were performed under ideal and non-ideal conditions of pH and temperature using serial 2-fold dilutions in 2.0% (*v*/*v*) blood cell suspension. The results obtained revealed impressive hemagglutinating activity in a wide pH range (4 ≤ pH ≤ 10; Table 1) evaluated at 20 °C, suggesting high lectin stability and predisposition to interact with sugars contained on erythrocyte walls. This protein activity was kept, even after a 1:2^6^ (*v*/*v*) dilution, corresponding to a specific activity of 27.83 HU mg^−1^, except for the two highest dilutions studied at pH = 10. Biomaterials with this profile are very important for developing high-tech analytical instrumentation, as they ensure greater robustness and allow the as-fabricated devices to be used in target samples without conventional preconditioning steps. Representative tests at pH < 4 or pH > 10 were not possible due to red blood cell hemolysis.

The ABL–enriched extract also showed remarkable thermostability, preserving the hemagglutinating activity when manipulated between 20 °C and 90 °C. Similar results were reported for lectins from *Agaricus arvensis* [44], *Macrophomina phaseolina* [47], and *Tricholoma mongolicum* [48] fungi. The thermal stability of glycoproteins is related to the types and proportion of carbohydrates in their structure, as high concentrations of them increase the intermolecular and intramolecular forces along the peptide chains and confer greater resistance to heat [49,50]. However, a continuous increase in temperature compromises the hemagglutinating activity by causing changes in the lectin secondary structure that, in turn, affect its ability to interact with red blood cells’ surface carbohydrates [50]. In addition, DSC data obtained with the purified ABL revealed a well-defined endothermic event around the identified critical temperature (Figure 2C), probably associated with lectin denaturation, supporting the arguments presented above.

### 3.3. Biosensor Manufacturing and Operation

**Figure 3:**
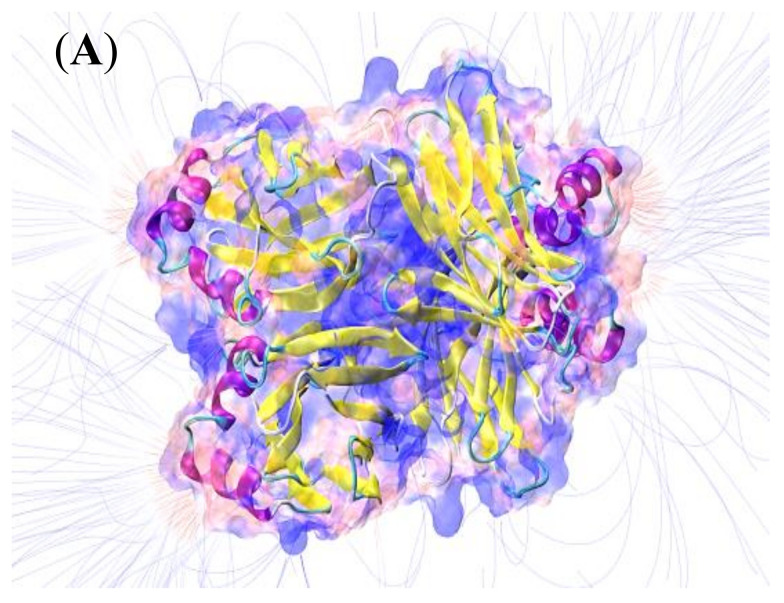

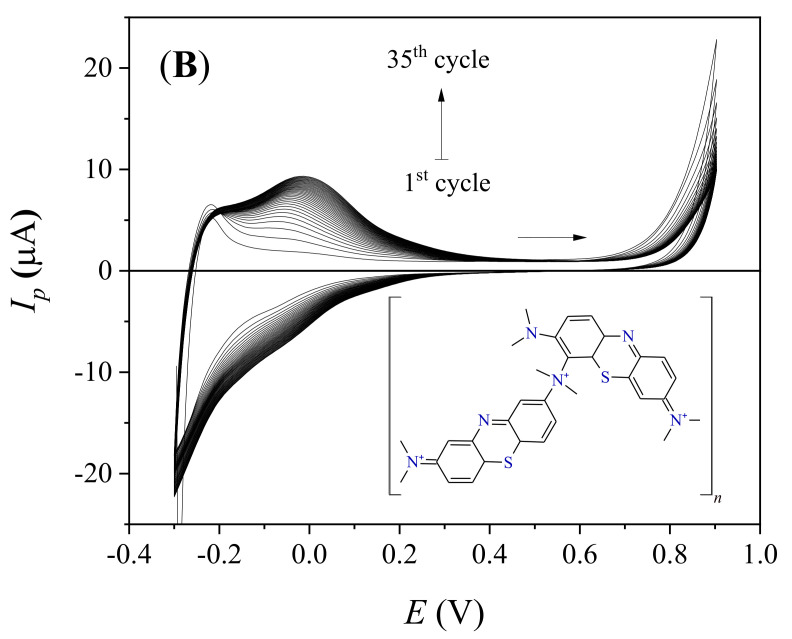
(**A**) ABL three–dimensional projection, highlighting the charge distribution and electric field around the molecule. (**B**) Cyclic voltammograms recorded during poly(methylene blue) electrodeposition on fluorine–doped tin oxide–coated glass at 50 mV s^−1^ using 100 mmol L^−1^ borate buffer (pH = 9.0) as the electrolyte. The insert illustrates the polymer structure.

Protein immobilization on electrode surfaces is among the most critical steps for developing biosensors, as the platform must simultaneously combine biocompatibility, response stability, and signal sensitivity in order to be operated under optimal conditions. Therefore, understanding the electrostatic characteristics of the working macromolecule is a fundamental condition, as it allows the most suitable electrode surfaces for the effective immobilization of ABL contained in the fungal extract to be identified. Computational quantum-chemistry results indicated that electrolytic environments with pH ≥ 6.7 (chosen working condition) provide an experimental condition above the pI of lectin isoforms, justifying the net negative charge concentrated in the α-helix regions (yellow segments of the peptide backbone illustrated in Figure 3A) and favoring ABL immobilization on positively charged surfaces. The data also revealed electric field signals around the entire molecular surface (surrounding blue lines), expanding the possibilities of interaction between the electrode material and target protein.

The electropolymerization of PMB on FTO represents a potential alternative to immobilize the selected lectin. The film of this conducting polymer is homogeneous, stable, and assumes a partial positive charge when produced from an alkaline electrolyte [51]. The cyclic voltammograms resulting from this process (Figure 3B), recorded at 50 mV s^−1^, showed the success in obtaining PMB/FTO, as well as the increase in film thickness as the number of cycles increased. When starting anodic polarization, a well-defined oxidation process was observed at *E_p_* = −0.22 V, resulting from the oxidation of methylene blue monomers pre-adsorbed on the electrode’s surface. At potentials above 0.70 V, a pronounced increase in current density occurred due to the formation of cation–radicals [52]. At least one amine group was expected to be oxidized in this process [27]. As cation–radicals are unstable, they irreversibly combine to form oligomeric species that precipitate on the electrode’s surface, leading to polymer film growth (representative structure in the insert of Figure 3B). This fact is directly related to the current density gain over the voltammetric cycles, continuous increase in a wider oxidation peak at *E_p_* = −0.01 V, and its corresponding reduction process at *E_p_* = −0.06 V.

**Figure 4:**
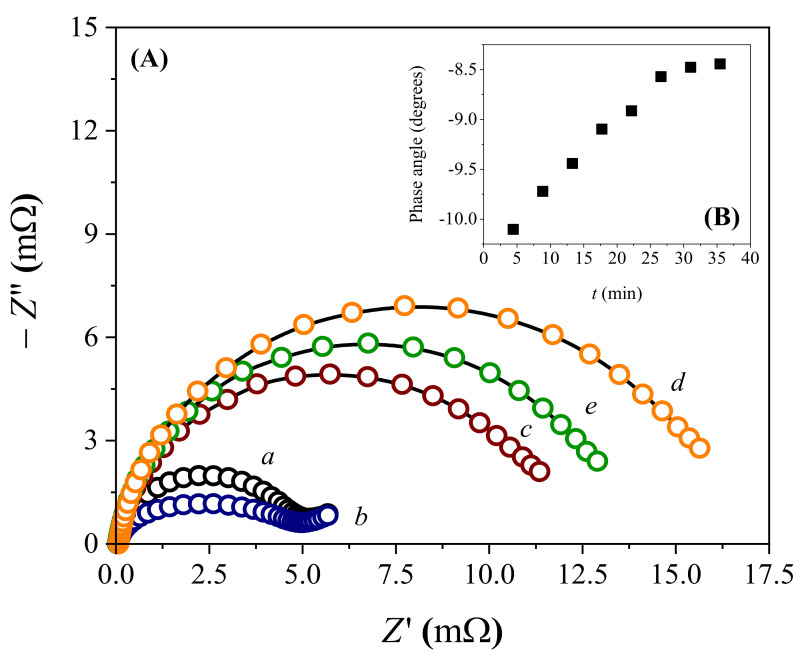
(**A**) Nyquist diagrams recorded with (*a*) fluorine–doped tin oxide–coated glass (FTO), (*b*) PMB/FTO, (*c*) ABL/PMB/FTO, (*d*) ABL/PMB/FTO, and 500 nmol L^−1^ lactose without irradiation, and (*e*) under blue LED influence with the following experimental conditions: 0.1 mmol L^−1^ K_3_[Fe(CN)_6_] as the redox probe, 50 mmol L^−1^ borate buffer as electrolyte, frequency ranging from 100 mHz to 100 kHz, and 5 mV modulation amplitude. (**B**) Effect of irradiation on the biosensor response over time using the same conditions stated above.

ABL immobilization on PMB/FTO was evaluated by EIS based on the *R_ct_* changes recorded between 100 mHz and 100 kHz using a 0.26 V redox potential and 5 mV modulation amplitude. From the Nyquist diagrams in Figure 4A, plotted by the relationship between the real (*Z*′) and imaginary (−*Z*″) impedances, it was noticed that both the FTO (*R_ct_* = 4.48 mΩ; line *a*) and PBM/FTO (*R_ct_ =* 3.82 mΩ; line *b*) had semicircles of reduced diameter, suggesting low charge-transfer resistance. The capacitive arc sizes were delimited by the relaxation of the electrical double layer, followed by the Warburg impedance associated with diffusional processes, fitting an equivalent circuit model illustrated in Figure S1—Supplementary Material. After modifying PMB/FTO with the ABL extract by drop-coating, there was a pronounced increase (*ca.* 2.4 to 2.8 times) in the capacitive arc radius (*R_ct_* = 10.8 mΩ; Figure 4A line *c*), as well as inhibition of the diffusional process, possibly caused by the successful immobilization of protein entities on the device’s electroactive surface. These results are well correlated with those obtained by cyclic voltammetry (Figure S2—Supplementary Material), which showed a reduction in current intensity after protein immobilization. The impedance experiments additionally showed a strong interaction between lactose and ABL/PMB/FTO, as indicated by the increase in the semicircle, even at low concentrations of this carbohydrate (*R_ct_* = 15.4 mΩ for 500 nmol L^−1^ lactose; Figure 4A line *d*). Similar results were observed with the isolated lectin, so the axial C-3 hydroxyl group in lactose was pointed out as the main ABL–carbohydrate binding site [15]. Such evidence confirms that *Agaricus bisporus* extract is a valuable biotechnological resource for lactose biosensing, adding value to the precursor biomaterial and avoiding complex, time-consuming, and expensive steps for protein purification.

Focusing on detection sensitivity and knowing that the polymer contained in the biosensor had a band–gap of 1.65 eV [27], the photosensitivity of the system under irradiation with a blue LED (470 nm; 10 W power) was evaluated. EIS data obtained over 1 h of irradiation showed a decay in the impedance values (*R_ct_* = 12.7 mΩ for 500 nmol L^−1^ lactose; Figure 4A line *e*) compared with those obtained in the absence of irradiation. The incident light reduced the recombination rate of photogenerated electron–hole pairs in the polymeric material, making interfacial phenomena more evident and improving the biosensor’s sensitivity. The photoelectrochemical effect reached the equilibrium condition after 30 min of continuous irradiation, as seen by the phase angle values in Figure 4B. This trend was also observed in the Bode plots shown in Figure S3—Supplementary Material. In this case, the ohmic resistance equilibrium was demonstrated by the gradual stabilization of the sigmoid curves formed between impedance and frequency, as well as the peak intensity resulting from the relationship between the phase angle and frequency. These profiles changed in intensity according to the capacitance of the electrical double layer, so the charge transfer became more deficient in the absence of irradiation.

### 3.4. Biosensor Analytical Performance

**Figure 5:**
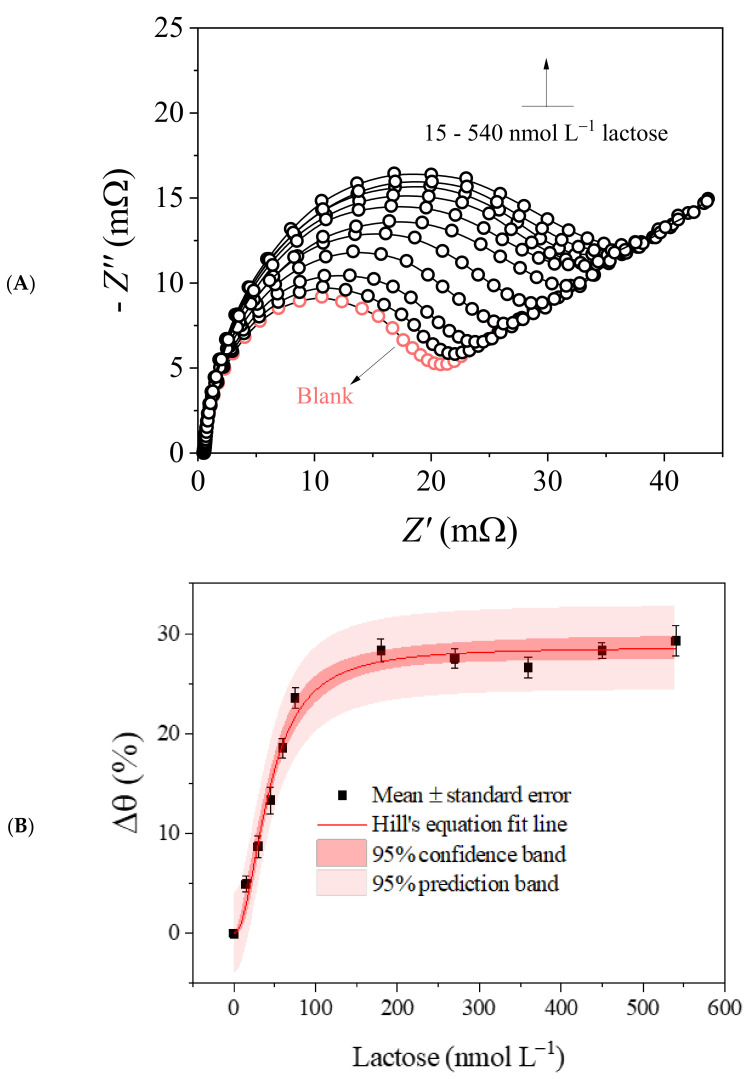
(**A**) Nyquist diagrams recorded for 15–540 nmol L^−1^ lactose using the ABL/PMB/FTO biosensor under blue LED irradiation, 0.1 mmol L^−1^ K_3_[Fe(CN)_6_] as the redox probe, and 50 mmol L^−1^ borate buffer as the electrolyte. (**B**) Dose–response curve and Hill’s sigmoidal fit for the corresponding photoelectrochemical analyses.

Based on the detected photoelectrochemical activity toward lactose, dose–response curves (15–540 nmol L^−1^ lactose; Figure 5A) were constructed from EIS measurements. The results obtained without irradiation can also be seen in Figure S4—Supplementary Material. The corresponding phase angle was recorded at 200 mHz, as the signal-to-noise ratio recorded under irradiation improved around this frequency. The saturation curve data were well-fitted by Hill’s equation (Figure 5B), considering $\Delta \theta_{max}$ = 0.11%, $K_{D}$ = 319.7, and *n* = 2. The mean values showed low dispersion and acceptable variance for 95% confidence, indicating high measurement precision. A low chi-square value (*χ*^2^ = 1.06 × 10^−5^) was also obtained, as well as a correlation coefficient close to unity (*R*^2^ = 0.9940), proving that the mathematical model explained the data variance well. Under the optimal analytical conditions, the ABL/PMB/FTO was able to detect lactose with LOD = 20.2 pmol L^−1^ and LOQ = 66.44 pmol L^−1^, as well as RSD < 5.4%, in the repeatability and reproducibility tests.

Without statistical fitting of the data to Hill’s model, it would be possible to work with different linear concentration ranges, but this procedure would make the dynamic range very narrow (saturation of the biosensor’s surface at [lactose] > 100 nmol L^−1^ was observed; Figure 5B), requiring sample dilution or pre-concentration steps that often lead to systematic errors in the results. Still, the sensitivity found with ABL/PMB/FTO was much higher than those reported for biosensing systems developed with cellobiose dehydrogenase (LOD = 3.5 mmol L^−1^ [53]), lactase (LOD = 100 µmol L^−1^ [54]), *β*-galactosidase/glucose oxidase (LOD = 0.23 µmol L^−1^ [28]), and *β*-galactosidase/glucose oxidase/peroxidase (LOD = 0.46 µmol L^−1^ [55]), although all demonstrated analytical efficiency for different purposes. The fitting of data to Hill’s equation further extended the biosensor’s applicability at higher concentrations.

Regarding the interference when the lactose content (10 nmol L^−1^) was measured in the presence of different monosaccharides (glucose, fructose, and maltose) or polysaccharides (sucrose, cellulose, and starch) prepared at the same analyte concentration, no signal influence was identified, indicating high response specificity and molecular selectivity. These data agree with the hemagglutination inhibition tests performed with the same carbohydrates. This profile was only possible due to the existence of binding sites in the protein structure capable of recognizing the axial hydroxyl group at the C-3 position of the carbohydrate with appreciable regioselectivity [12,15]. The dissociation constant value was relatively low, and Hill’s coefficient suggested at least two domains of ABL–lactose interaction. Intraday and interday repeatability experiments performed for 10 nmol L^−1^ lactose with a single device (RSD < 6.1%), along with reproducibility measurements performed with three different devices (RSD < 7.3%), showed that the results obtained were precise. As for stability, considering six different measurements of the aforementioned carbohydrate concentrations performed with a single device over three months, almost 93.3% of the initial electroanalytical signal was maintained. Therefore, ABL/PMB/FTO has great potential for the analysis and quality control of lactose in samples whose concentration of this carbohydrate is critical for the reaction of interest.

### 3.5. Real Application and Validation

**Figure 6:**
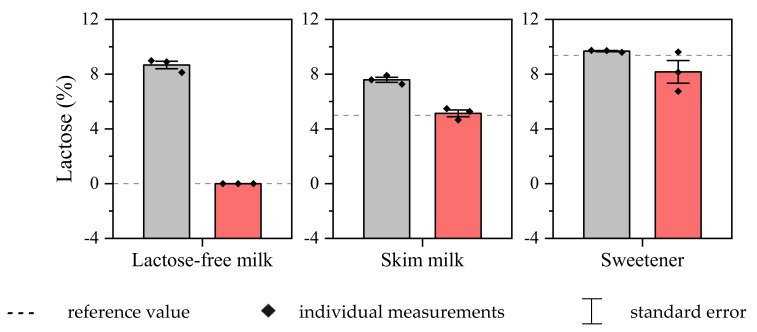
Results obtained for lactose analysis in commercial food products using the photoelectrochemical (red bars) and spectrometric (gray bars) procedures.

The procedure developed with the photoelectrochemical biosensor was adapted for the detection and quantification of lactose in commercial samples of skimmed milk (6.0%; *m*/*v*), lactose-free milk, and sweetener (9.0%; *m*/*v*). The results were consistent with the information from the manufacturers (92.1–102.7% recovery), confirming the successful application of ABL/PMB/FTO for the inspection of the studied commercial products. The results were compared with those obtained by the standard spectrophotometric method for reducing sugars, based on the reaction with 3,5-dinitrosalisylic acid [39], showing good correlation and low error margin between triplicate measurements (Figure 6). Another important advantage of the biosensor was its selectivity for analyzing lactose-free milk, as the spectrophotometric method does not distinguish between different reducing sugars present in the sample, generating false positive results. In other words, ABL/PMB/FTO provided reliable results for the presence of lactose in the studied samples, even when associated with different excipients, proving the feasibility of the proposed photoelectrochemical method.

## 4. Conclusions

*Agaricus bisporus* mushroom lectin, here called ABL, has a strong affinity for lactose, allowing its screening through a highly specific and selective mechanism. However, the long time required to isolate and purify this protein, together with the reduced hemagglutinating activity in relation to the crude extract, showed that the latter can be more advantageous for developing high-performance biosensors. The protein extract of the macrofungus still conserved lectin activity over wide pH and temperature ranges, avoiding the time-consuming steps of protein isolation and purification while making its manipulation cheaper and simpler. Theoretical and experimental studies indicated that the PMB/FTO electrode surface was electrostatically propitious and biocompatible for immobilizing ABL from mushroom extract, as well as for increasing the sensitivity of lactose biorecognition by photoelectrochemical stimulation. The ABL/PMB/FTO biosensor also showed remarkable signal stability, measurement accuracy, and ability to analyze lactose in commercial products, even in the presence of various excipients. Additionally, this study adds value to *Agaricus bisporus* biomass as a biomaterial with enormous biotechnological potential, driving further research on this matter.

## Figures and Tables

**Table 1:** Hemagglutinating activity induced by ABL–based extract after serial 2–fold dilutions in PBS (4 ≤ pH ≤ 10). Images were captured after shaking the reaction wells.

pH	Hemagglutinating Activity (HU)					
	Control *	4	8	16	32	64
4	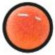	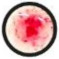	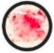	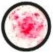	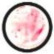	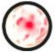
	−	+	+	+	+	+
6	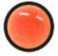	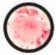	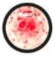	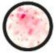	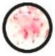	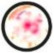
	−	+	+	+	+	+
7	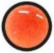	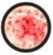	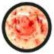	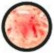	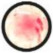	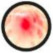
	−	+	+	+	+	+
8	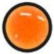	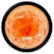	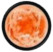	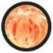	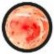	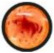
	−	+	+	+	+	+
10	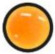	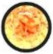	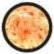	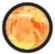	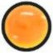	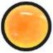
	−	+	+	+	−	−

* 2.0% (*v*/*v*) blood cell suspension.

## Data Availability

Not applicable.

## Supplementary Materials

The following supporting information can be downloaded at: https://www.mdpi.com/article/10.3390/bios13020224/s1, Figure S1: Equivalent circuit used to fit impedance data, composed of electrolyte resistance (Rs), polarization resistance (Rct), Warburg impedance (W) and system capacitance (CPE); Figure S2: Cyclic voltammograms registered for 0.1 mmol L^−1^ K3[Fe(CN)6] on a) FTO, (b) PMB/FTO and (c) ABL/PMB/FTO at 50 mV s^−1^, using 50 mmol L^−1^ borate buffer (pH = 9.0) and 50 mmol L^−1^ KCl as electrolyte. The insert illustrates the cyclic voltammograms obtained in the absence of the redox probe; Figure S3: Bode plots referring to the ABL/PMB/FTO signal stabilization for 500 nmol L^−1^ lactose under blue LED irradiation over 40 min, using the following experimental conditions: 0.1 mmol L^−1^ K3[Fe(CN)6] as redox probe; 50 mmol L^−1^ borate buffer as electrolyte; frequency ranging from 100 mHz to 100 kHz; and 5 mV modulation amplitude; Figure S4: Nyquist diagrams recorded (a) without lactose and containing (b) 100, (c) 200, (d) 300, (e) 400 and (f) 500 nmol L^−1^ of this carbohydrate, using the ABL/PMB/FTO biosensor without irradiation, 0.1 mmol L^−1^ K3[Fe(CN)6] as redox probe, and 50 mmol L^−1^ borate buffer as electrolyte.
