# Supplementary material for: Agaricus bisporus Wild Mushroom Extract as Lectin Source for Engineering a Lactose Photoelectrochemical Biosensor
Source: Biosensors (Basel). 2023 Feb 3;13(2):224. doi: 10.3390/bios13020224 (PMC9953549; doi:10.3390/bios13020224)
Supplement: Supplementary file 1 [file biosensors-13-00224-s001.zip › biosensors-2147895-supplementary.pdf]

Supplementary Material

Agaricus bisporus wild mushroom extract as lectin source for engineering a lactose photoelectrochemical biosensor

André O. Santos [1], Vanessa E. Abrantes-Coutinho [1], Simone Morais [2] and Thiago M.B.F. Oliveira [1,*]

[1] Centro de Ciência e Tecnologia, Universidade Federal do Cariri, 63048-080 Juazeiro do Norte, CE, Brazil
[2] REQUIMTE-LAQV, Instituto Superior de Engenharia do Porto, Instituto Politécnico do Porto, Rua Dr. António Bernardino de Almeida, 431, 4249-015 Porto, Portugal
* Correspondence: thiago.mielle@ufca.edu.br

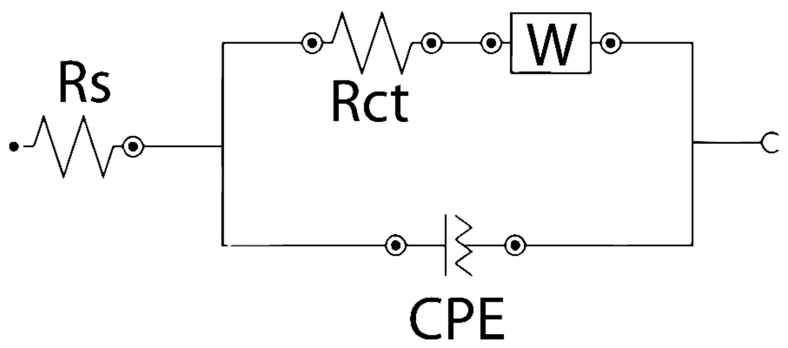

Figure S1. Equivalent circuit used to fit impedance data, composed of electrolyte resistance (Rs), polarization resistance (Rct), Warburg impedance (W) and system capacitance (CPE).

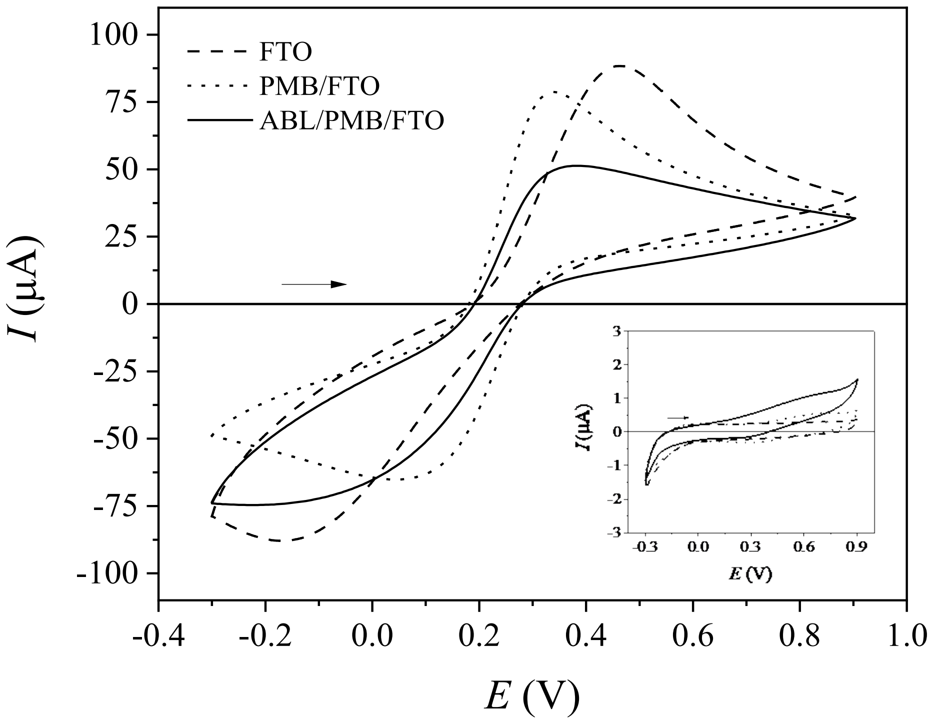

Figure S2. Cyclic voltammograms registered for 0.1 mmol L^{-1} $K_3[Fe(CN)_6]$ on *a*) FTO, (*b*) PMB/FTO and (*c*) ABL/PMB/FTO at 50 mV s^{-1}, using 50 mmol L^{-1} borate buffer (pH = 9.0) and 50 mmol L^{-1} KCl as electrolyte. The insert illustrates the cyclic voltammograms obtained in the absence of the redox probe.

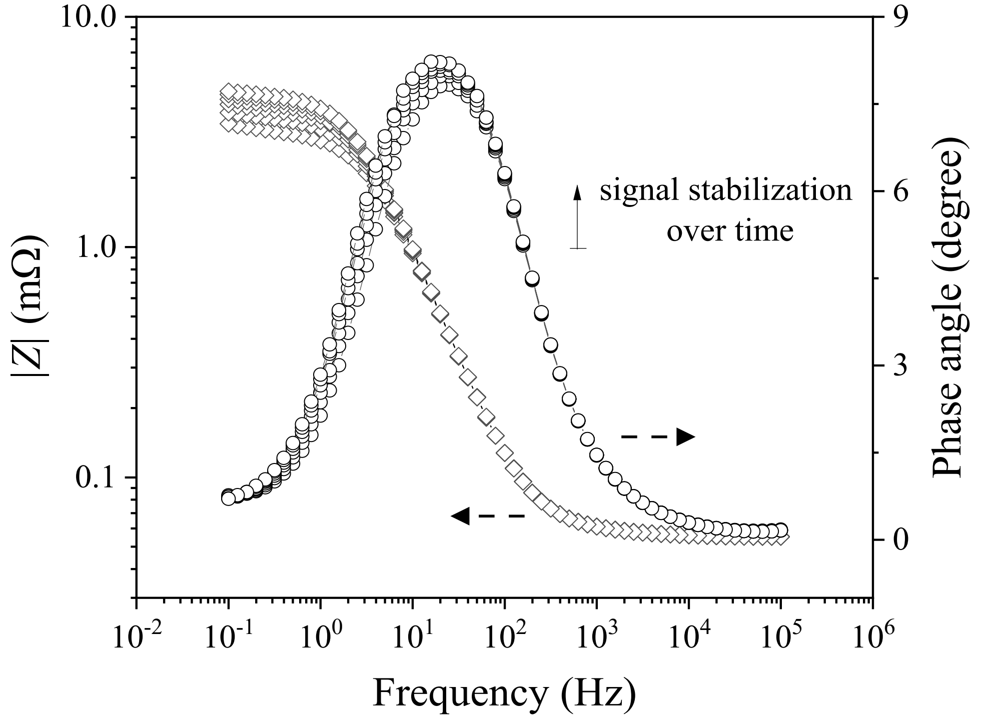

Figure S3. Bode plots referring to the ABL/PMB/FTO signal stabilization for 500 nmol L^{-1} lactose under blue LED irradiation over 40 min, using the following experimental conditions: 0.1 mmol L^{-1} $K_3[Fe(CN)_6]$ as redox probe; 50 mmol L^{-1} borate buffer as electrolyte; frequency ranging from 100 mHz to 100 kHz; and 5 mV modulation amplitude.

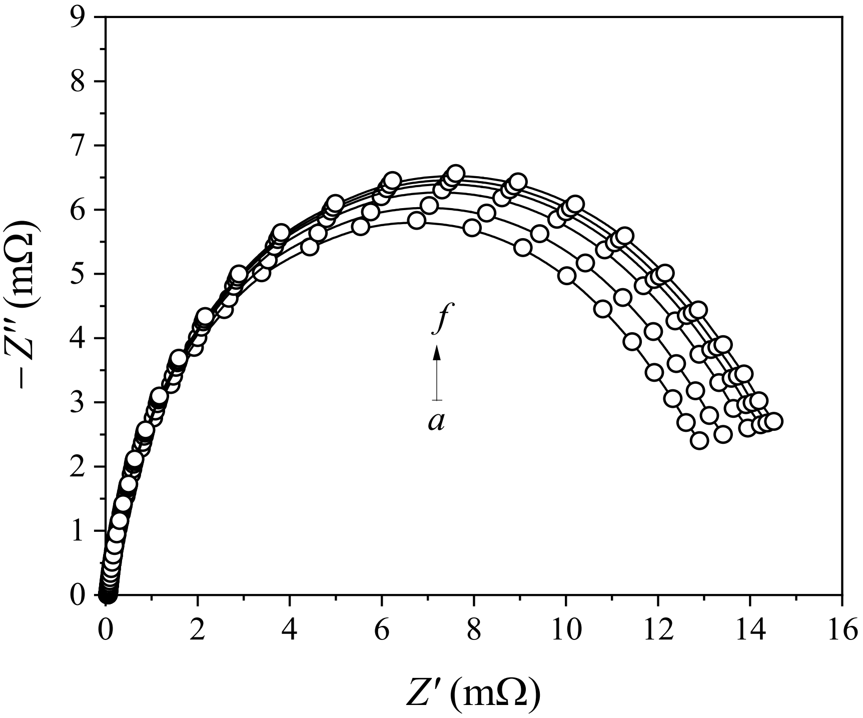

Figure S4. Nyquist diagrams recorded (*a*) without lactose and containing (b) 100, (c) 200, (d) 300, (e) 400 and (f) 500 nmol L^{-1} of this carbohydrate, using the ABL/PMB/FTO biosensor without irradiation, 0.1 mmol L^{-1} $K_3[Fe(CN)_6]$ as redox probe, and 50 mmol L^{-1} borate buffer as electrolyte.
